# Evaluating the effect of tanning response to sun exposure on the risk of skin diseases through Mendelian randomization

**DOI:** 10.3389/fgene.2022.967696

**Published:** 2022-09-02

**Authors:** Weidong Ping, Qiming Zhao, Shuhong Ge, Xin Wang, Fei Li, Xiaoxiang Huang

**Affiliations:** Department of Plastic Surgery, Zhejiang Hospital, Hangzhou, Zhejiang, China

**Keywords:** tanning, sun exposure, skin diseases, Mendelian randomization, melanin pigmentation

## Abstract

**Background:** Until now, the relevance of the tanning response to sun exposure and skin diseases has incomplete and inconsistent epidemiological observations. In this case, it is valuable to find out the causality of tanning response to sun exposure and skin diseases, and take a step further toward developing effective therapies as well as prevention methods.

**Methods:** We investigated the causal effect of tanning response to sun exposure on 10 major skin diseases that have been studied in recent large-scale genome-wide association studies (GWASs). Significant independent genetic variants from large-scale GWAS on ease of skin tanning (N = 453,065) are selected as the effective instrumental variables (IVs). For each skin disease, we extracted the summary statistics of those IVs (or their proxies) from the corresponding skin disease-GWAS as the valid IVs. Mendelian randomization (MR) was further performed to evaluate the causal association of ease of skin tanning with each of the skin diseases using different statistical methods, including inverse-variance weighted (IVW), the weighted median, and MR-Egger. Sensitivity analysis was also conducted to evaluate the effect of horizontal pleiotropy and heterogeneity.

**Results:** We observe significant associations between six skin diseases with tanning response to sun exposure with adjusted *p*-value derived by IVW less than 0.05 and with nominal *p* value less than 0.05 at the same time derived by either MR-Egger or weighted median. The six skin diseases include actinic keratosis (IVW FDR = 1.71E-40, MR Egger *p*-value = 3.46E-22), seborrhoeic keratosis (IVW FDR = 2.97E-4, MR Egger *p*-value = 1.06E-3), blepharochalasis (IVW FDR = 1.30E-3, MR Egger *p*-value = 2.91E-4), seborrhoeic dermatitis (IVW FDR = 1.29E-2, MR Egger *p*-value = 1.23E-2), malignant melanoma of skin (IVW FDR = 2.95E-2, MR Egger *p*-value = 1.91E-2), and freckles (IVW FDR = 2.95E-2, weighted median *p*-value = 1.02E-3). Interestingly, we find increased trends of developing all of the six skin diseases with increased tanning response to sun exposure (beta values are positive using IVW, MR-egger, and weighted median methods). We also replicate the association on three skin diseases using an independent outcome GWAS cohort, including malignant melanoma of the skin (replication IVW *p*-value = 2.13E-39), actinic keratosis (replication IVW *p*-value = 4.64E-32), and seborrhoeic keratosis (replication IVW *p*-value = 1.79E-3).

**Conclusion:** Our observation shows that the tanning response to sun exposure is positively correlated with the development of skin diseases in people of European descent by Mendelian randomization studies. But randomized controlled trials are still needed to add proof to our observations.

## 1 Introduction

Skin disease is one of the most common human diseases across cultures, ages, and races. There is a significant public health problem associated with inadequate sun exposure. According to studies conducted over the past decade, insufficient sun exposure may be responsible for 340,000 deaths in the United States and 480,000 deaths in Europe each year, and an increased incidence of breast cancer, colorectal cancer, hypertension, cardiovascular disease, metabolic syndrome, multiple sclerosis, Alzheimer’s disease, autism, asthma, type 1 diabetes, and myopia (Alfredsson et al., 2020). It is widely acknowledged that many skin diseases are associated with tanning and respond to sun exposure (Visconti et al., 2018).

It has been reported that the change from a subarctic/temperate to a subtropical climate for 4 weeks improved significantly skin symptoms and quality of life in children, even for 3 months after the return. Until now, sun exposure has not been studied in adult patients with atopic dermatitis (Napolitano et al., 2021). However, the deterioration of the ozone layer and the increasing use of sun tanning beds have led to an increase in skin-damaging ultraviolet radiation (Zitás and Mészáros, 2016). Among the most probable mechanisms of contamination-induced dermatological hazards are oxidative stress, inflammation, and metabolic impairment. This stress will be further aggravated by the deleterious synergy between pollution and sunlight. Some experiments identified a few polycyclic aromatic hydrocarbons (PAHs) inducing huge toxic stress, at nanomolar concentrations, when exposed to long ultraviolet radiation A (UVA) wavelengths (Marrot, 2018). Tanning response to sun exposure is the process of melanin pigmentation, which is a skin-protecting response from DNA photodamage (Ortonne, 1990; Gilchrest and Eller, 1999). Nine major skin diseases or traits are under investigation in our study, including malignant melanoma of the skin, actinic keratosis, blepharochalasis, seborrhoeic keratosis, and melanocytic naevi, scne vulgaris, freckles, lichen simplex chronicus, and prurigo and chalazion. The World Health Organization(WHO) declared having sufficient evidence to classify exposure to ultraviolet radiation (sun exposure) as carcinogenic. Also, sun exposure is consistently associated with malignant melanoma (Lucas et al., 2014; Raimondi et al., 2020). Actinic keratosis (AK), a lesion that will easily develop into malignancy, is also caused by excessive exposure to solar radiation (Berman and Cockerell, 2013). Seborrhoeic keratosis is reported to associate with sun exposure and tanning (Del Rosso, 2017). Sunlights have some influence on the occurrence of Melanocytic naevi (Barsoum and Harrison, 2020). The common naevi distribution pattern over the skin is consistent with the sun exposure habit (Carli et al., 1998). As for Acne vulgaris, its pathogenesis is various and complicated but sunlight could affect acne vulgaris negatively (Amblard and Leccia, 1992). For freckles, the common name of ephelides and lentigines, the causes are not completely clear and influenced by both genetic and environmental factors, but for sure, sunlight is a trigger (Passeron and Picardo, 2018). While blepharochalasis, a rare eyelid disorder has no confirmed etiology, so does the sun exposure factor. Also, lichen simplex chronicus (LSC) is a multifactor induced skin disorder, and clinical manifestations are reginal skin thickening by frequently rubbing or scratching; sunlight factor is not reported to be associated with this disorder till now. Also, prurigo is an inflammatory skin disease characterized by intensely pruritic, causes unclear, but most likely related to hypersensitivity, neuropsychiatric factors, and genetic allergic constitution; sun exposure influence is not reported. Also, chalazia are caused by inflammation and obstruction of the eyelids’ sebaceous glands (Taylor and Sober, 1996).

The causal link between tanning response to sun exposure and developing skin diseases remains incomplete and inconsistent. Therefore, in this study, we investigated the association between sun exposure and 10 major skin diseases or traits using Mendelian randomization (MR). Mendelian randomization design has been widely applied in recent years to determine the causal inferences of its significant advantage of overcoming the methodological limitations of observational studies. In this study, we used MR study to explore the causal association between tanning response to sun exposure (exposure) and skin diseases (outcomes) in European people of descent by multiple large-scale genome-wide association study (GWAS) datasets. The outcomes under investigation include acne vulgaris, actinic keratosis, blepharochalasis, chalazion, freckles, malignant melanoma of the skin, melanocytic naevi, prurigo nodularis, seborrhoeic dermatitis, and seborrhoeic keratosis.

## 2 Materials and methods

### 2.1 Study design

MR analysis used a group of single nucleotide polymorphisms (SNPs) as instrumental variables (IVs) to infer the causal effect of an exposure on an interesting outcome. The genome-wide association study (GWAS) summaries of exposure and various outcomes were downloaded from the MRC IEU OpenGWAS database. We extracted GWAS summary statistics of ease of skin tanning trait as the exposure data and 10 skin diseases as outcomes including acne vulgaris, actinic keratosis, blepharochalasis, chalazion, freckles, malignant melanoma of the skin, melanocytic naevi, prurigo nodularis, seborrhoeic dermatitis, and seborrhoeic keratosis.

**FIGURE 1:**
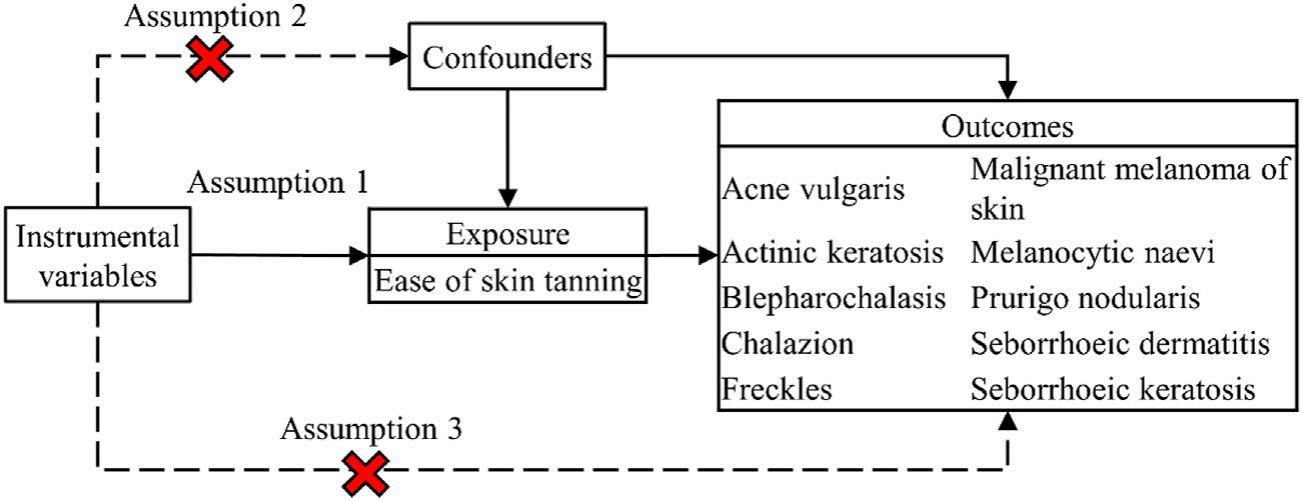
The flow chart of the MR study.

In general, MR analysis needs to meet three assumptions: 1) the IVs should be significantly associated with the exposure; 2) the IVs should be independent. and 3) the IVs should not affect the outcome except through exposure. The overall design of this study is shown in Figure 1.

### 2.2 Selection of genetic variants associated with tanning response to sun exposure

The genetic associations on tanning response to sun exposure are derived from a large genome-wide association study of ease of skin tanning in 453,065 subjects of European ancestry (https://gwas.mrcieu.ac.uk/datasets/).

The cohort used in the GWAS analysis is from the UK Biobank (UKBB), which is a prospective cohort study that covered more than 500,000 individuals from the United Kingdom, aged from 40 to 70. Biological samples, including blood, urine, and saliva were collected and every participant accepted an extensive questionnaire investigation about health conditions and lifestyle. In accordance with the Declaration of Helsinki, every participant signed informed consent.

The UKBB study collected individual self-report about the ease of skin tanning of European ethnicity. The individuals who reported never tanned only burn, or get mildly or occasionally tanned, were included in the group of low tan responses. On the contrary, people who reported showing moderate or severe tanning were included in the group of high tan responses. Logistic regression was using PLINK assuming an additive genetic model and taking sex as a covariate, as well as the first five principal components evaluated on the genomic data, to control the potential population stratification.

We retrieved the GWAS dataset of ease of skin tanning from the MRC IEU GWAS database, where 9,851,867 SNPs across the genome were used for association analysis. The LD score regression analysis was used to estimate the SNP heritability for tanning response to sun exposure, and the total observed scale H2 is 0.1917 (se = 0.06), with lambda GC equal to 1.43. The GWAS variants with a *p*-value less than 5E-8 were used as potential instruments in the following MR studies after LD pruning.

### 2.3 GWAS datasets on skin diseases in discovery and replication phases

We investigated the causal effect of sun exposure on 10 major skin diseases, including acne vulgaris, actinic keratosis, blepharochalasis, chalazion, freckles, malignant melanoma of the skin, melanocytic naevi, prurigo nodularis, seborrhoeic dermatitis, and seborrhoeic keratosis. The demographic profiles of GWAS cohorts related to these diseases in the discovery phase are shown in the Results section. The GWASs on all skin diseases were performed in the European population, except that GWAS on freckles was performed in the East Asian population because the freckles-related GWAS for the European population is not available in the IEU database. For each skin disease, we extracted the summary statistics of those IVs (or their proxies) from the corresponding skin disease-GWAS to perform MR analysis.

In the discovery phase, the GWASs of the 10 investigated skin diseases were derived from the FinnGen biobank (https://www.finngen.fi/fi), which has collected biological samples from 500,000 participants in Finland over 6 years. To ensure the robustness of our discovery, we replicated 5 MR studies using UKBB cohorts, including actinic keratosis, chalazion, malignant melanoma of the skin, melanocytic naevi, and seborrhoeic keratosisdata, while the other five skin diseases lack necessary GWAS summaries in the UKBB.

### 2.4 MR analysis

Three MR analysis methods were chosen in this study, including the inverse-variance weighted (IVW) (Bowden et al., 2017), the weighted median (Bowden et al., 2016), and the MR-Egger test (Bowden et al., 2015). The effect size (beta) corresponds to one standard deviation (SD) in tanning response to sun exposure. All statistical tests were performed with R Packages TwoSampleMR (Hemani et al., 2018). The resulting *p* values of IVW were corrected by Benjamini–Hochberg (BH) procedure. Under the threshold of IVW-adjusted *p*-value < 0.05, either nominal *p*-value of MR Egger <0.05 or nominal *p*-value of weighted median <0.05, we declared significant MR results. The technical details of the three methods are described below.

#### 2.4.1 IVW method

The inverse-variance weighted (IVW) method uses a weighted linear regression of SNP-exposure coefficients and SNP-disease coefficients to estimate the effect of exposure on outcome. Suppose there are N participants and each participant is indexed by i . Let G represent J genetic variants ( $G_{1},\;\ldots ,G_{J}$ ), X represent exposure, Y represent outcome, $\gamma_{j}$ represent the effect of $G_{j}$ on the exposure and $\alpha_{j}$ represent the effect of $G_{j}$ on the outcome. The exposure can be considered as the result of linear regression of genetic variants $G_{j}$ , confounder U , and the independent error term $\varepsilon_{X}$ . The outcome can be seen as a result of linear regression of the exposure X , genetic variants $G_{j}$ , confounder U , and independent error term $\varepsilon_{Y}$ . Let β represent the causal effect of the exposure on the outcome; these can be written as follows:

$$X_{i}=\sum_{j=1}^{J}\gamma_{j}G_{ij}+U_{i}+\varepsilon_{X_{i}},$$ (1)

$$Y_{i}=\sum_{j=1}^{J}\alpha_{j}G_{ij}+\beta X_{i}+U_{i}+\varepsilon_{Y_{i}}.$$ (2)

In MR analysis, genetic variants need to meet the following three assumptions to be valid instrumental variables (IVs): 1) the IVs should be significantly associated with the exposure; 2) the IVs should be independent; and 3) the IVs should not affect the outcome except through the exposure.

The IVW method is efficient when all genetic variants are valid IVs, which implies that $\gamma_{j}\neq 0$ in Eq. 1 to satisfy assumption 1) and $\alpha_{j}=0$ in Eq. 2 to satisfy assumption (2).

Let $\Gamma_{j}$ represent the coefficient from a regression of the outcome on the genetic variant $G_{j}$ . Eq. 2 can be simplified as

$$Y_{i}=\Gamma_{j}G_{ij}+\varepsilon_{Y_{i\;}\;},$$ (3)

$$=\left(\alpha_{j}+\beta \gamma_{j}\right)G_{ij}+\varepsilon_{Y_{i}}.$$ (4)

If the genetic variant is valid IV, $\alpha_{j}=0$ , then,

$${\hat{\beta }}_{j}=\frac{{\hat{\Gamma }}_{j}}{{\hat{\gamma }}_{j}}.$$ (5)

The causal effect can be estimated from GWAS using inverse-variance weighted (IVW) as follows:

$${\hat{\beta }}_{IVW}=\frac{\sum_{j=1}^{J}{\hat{\gamma }}_{j}^{2}\sigma_{Y_{j}}^{-2}{\hat{\beta }}_{j}}{\sum_{j=1}^{J}{\hat{\gamma }}_{j}^{2}\sigma_{Y_{j}}^{-2}},$$ (6)

where $\sigma_{Y_{j}}$ is the standard error in the regression of the outcome on the $G_{j}$ .

#### 2.4.2 MR-egger method

The MR-Egger method is an alternative method for Mendelian randomization analysis which is like the IVW method. In MR-Egger, ${\hat{\Gamma }}_{j}$ consists of two β items:

$${\hat{\Gamma }}_{j}=\beta_{0E}+\beta_{E}{\hat{\gamma }}_{j}.$$ (7)

In comparison with the IVW method, MR-Egger has a more intercept term, $\beta_{0E}$ . The value of ${\hat{\beta }}_{0E}$ is used to measure the average pleiotropy across SNPs. If all SNPs are valid IVs, then $\beta_{0E}$ = 0, and the estimated ${\hat{\beta }}_{E}$ will equal the estimation of the IVW method. If the intercept term differs from zero, it indicates the presence of overall directional pleiotropy and the IVW estimate is biased at this time. The MR-Egger estimation for $\beta_{E}$ and ${\hat{\beta }}_{E}$ , is consistent even if all SNPs are invalid, which is more robust.

#### 2.4.3 Weighted median method

Let ${\hat{\beta }}_{\left(1\right)},\ldots ,\;{\hat{\beta }}_{\left(J\right)}$ be the J casual effect estimating which ordered from the smallest ( ${\hat{\beta }}_{\left(1\right)}$ ) to the largest ( ${\hat{\beta }}_{\left(J\right)}$ ). Define $w_{j}$ is the inverse variance of ${\hat{\beta }}_{\left(j\right)}$ and $S_{j}=\overset{j}{\underset{k=1}{\sum }}w_{k}$ . Each $w_{j}$ is standardized and the sum of $w_{j}$ , $S_{J}$ equals 1. Let $p_{j}=100\left(S_{j}-\frac{w_{j}}{2}\right)th$ and $p_{j}$ represent the quantile. The casual effect estimated through the weighted median method, ${\hat{\beta }}_{WM}$ , is defined as the 50th percentile of the distribution. If at least 50% of the $w_{j}$ comes from valid IVs, the weighted median method provides a valid estimate of the casual effect.

### 2.5 Pleiotropy analysis

The pleiotropy analysis is on the ground of the MR-Egger intercept test.

In addition, we performed a heterogeneity test with Cochran’s Q statistic (together with the $I^{2}$ statistic) from IVW, which may indicate the existence of heterogeneity caused by pleiotropy or other causes. In general, Cochran’s Q statistic follows a $x^{2}$ distribution with *k*-1 degrees of freedom (where *k* stands for the number of genetic variants). $I^{2}=\left(Q-\left(k-1\right)\right)/Q\times 100\%$ ranges from 0 to 100%, and 0–25%, 25–50%, 50–75%, and 75–100% corresponding to low, moderate, large, and extreme heterogeneity, respectively. All statistical tests were performed with R Package TwoSampleMR.

## 3 Results and discussion

We performed MR analyses to measure the causal effect of tanning response to sun exposure on 10 common skin diseases. The demographic profiles of GWAS cohorts related to these diseases in the discovery phase are shown in Table 1. The GWASs on all skin diseases were performed in the European population, except that GWAS on Freckles was performed in the East Asian population because the freckles-related GWAS for the European population is not available in the IEU database. For each skin disease, the number of IVs represents the number of SNPs as IVs (or as IVs’ proxies with LD R2 > 0.4) shown in the corresponding skin disease-GWAS. In the discovery phase, the GWASs of the nine investigated skin diseases were derived from the FinnGen biobank (https://www.finngen.fi/fi).

**TABLE 1:** Summary of the outcome GWAS in the discovery study.

Outcome trait	ID in the IEU GWAS database	Population	Number of IVs	Number of	
				Cases	Controls
Acne vulgaris	finn-b-L12_ACNE_VULGARIS	European	125	1,092	211,139
Actinic keratosis	finn-b-L12_ACTINKERA	European	125	4,817	213,273
Blepharochalasis	finn-b-H7_BLEPHAROCHALASIS	European	125	4,135	203,231
Chalazion	finn-b-H7_CHALAZION	European	125	1,777	203,231
Malignant melanoma of skin	finn-b-C3_MELANOMA_SKIN	European	125	98	218,694
Melanocytic naevi	finn-b-CD2_BENIGN_MELANOCYTIC	European	125	4,035	214,757
Prurigo nodularis	finn-b-L12_PRURIGONOD	European	125	309	198,740
Seborrhoeic dermatitis	finn-b-L12_SEBORRHOEIC	European	125	1,253	198,740
Seborrhoeic keratosis	finn-b-L12_SEBORRKERAT	European	125	2,434	207,482
Freckles	ebi-a-GCST006091	East Asian	35	7,148	4,034

The MR analyses are performed using three approaches (inverse variance weighted approach, MR Egger approach, and weighted median approach), and the overall results are presented in Table 2. Under the threshold of IVW FDR <0.05, and either nominal *p*-value of MR Egger <0.05 or nominal *p*-value of weighted median <0.05, we declare significant MR results. As a result, six of ten skin diseases we examined are significantly associated with the tanning response to sun exposure. In addition, we conduct sensitivity analyses and the results are given in Table 3. In the following results, we will discuss the effect of tanning response to sun exposure on skin diseases that passed the significance threshold.

**TABLE 2:** MR results between tanning response to sun exposure and ten skin diseases in the discovery study.

Outcome trait	IVW				MR Egger			Weighted median		
	Beta	Se	*p*-value	FDR	Beta	se	*p*-value	Beta	se	*p*-value
Actinic keratosis	1.19	0.09	1.71E-41	1.71E-40	1.28	0.11	3.46E-22	1.33	0.09	2.39E-45
Seborrhoeic keratosis	0.28	0.07	5.94E-05	2.97E-04	0.29	0.09	1.06E-03	0.27	0.11	1.30E-02
Blepharochalasis	0.22	0.06	3.90E-04	1.30E-03	0.28	0.08	2.91E-04	0.26	0.09	2.84E-03
Seborrhoeic dermatitis	0.29	0.10	5.15E-03	1.29E-02	0.32	0.12	1.23E-02	0.52	0.15	3.40E-04
Malignant melanoma of skin	0.83	0.35	1.67E-02	2.95E-02	1.01	0.42	1.91E-02	0.63	0.51	2.12E-01
Freckles	1.01	0.42	1.77E-02	2.95E-02	0.93	0.66	1.69E-01	0.77	0.23	1.02E-03
Melanocytic naevi	0.12	0.07	6.04E-02	8.62E-02	0.09	0.08	2.55E-01	0.19	0.09	3.02E-02
Chalazion	0.14	0.08	9.83E-02	1.23E-01	0.14	0.10	1.63E-01	0.08	0.12	5.05E-01
Acne vulgaris	-0.06	0.10	5.43E-01	6.03E-01	-0.13	0.12	3.06E-01	-0.07	0.16	6.49E-01
Prurigo nodularis	0.05	0.19	8.15E-01	8.15E-01	0.09	0.24	7.14E-01	0.14	0.28	6.16E-01

Abbreviations: se, standard error; IVW, inverse variance weighted; MR, mendelian randomization; FDR, false discovery rate adjusted *p* value.

**TABLE 3:** The results of sensitivity analyses in the discovery MR study.

Outcome	Horizontal pleiotropy			Heterogeneity					
	Egger intercept	se	*p*-value	IVWQ	IVW Q df	IVW *p*-value	MR Egger Q	MR Egger Q df	MR Egger *p*-value
Actinic keratosis	−7.69E-03	5.35E-03	0.15	367.34	124	6.41E-26	361.28	123	2.82E-25
Seborrhoeic keratosis	−5.50E-04	4.34E-03	0.90	131.74	124	3.00E-01	131.73	123	2.79E-01
Blepharochalasis	−5.45E-03	3.86E-03	0.16	168.62	124	4.77E-03	165.93	123	5.99E-03
Seborrhoeic dermatitis	−2.78E-03	6.32E-03	0.66	150.25	124	5.44E-02	150.01	123	4.93E-02
Malignant melanoma of the skin	−1.56E-02	2.15E-02	0.47	138.46	124	1.77E-01	137.87	123	1.70E-01
Freckles	3.52E-03	2.33E-02	0.88	227.28	34	1.91E-30	227.12	33	7.68E-31
Melanocytic naevi	2.79E-03	4.03E-03	0.49	191.57	124	9.46E-05	190.83	123	8.60E-05
Chalazion	−4.82E-04	5.23E-03	0.93	143.73	124	1.09E-01	143.72	123	9.76E-02
Acne vulgaris	5.77E-03	6.23E-03	0.36	113.06	124	7.50E-01	112.20	123	7.48E-01
Prurigo nodularis	−3.68E-03	1.20E-02	0.76	134.06	124	2.53E-01	133.96	123	2.35E-01

Abbreviations: se, standard error; IVW, inverse variance weighted; MR, mendelian randomization; Q, Cochran’s Q test estimate; df, Cochran’s Q test degrees of freedom.

### 3.1 Effect on actinic keratosis

**FIGURE 2:**
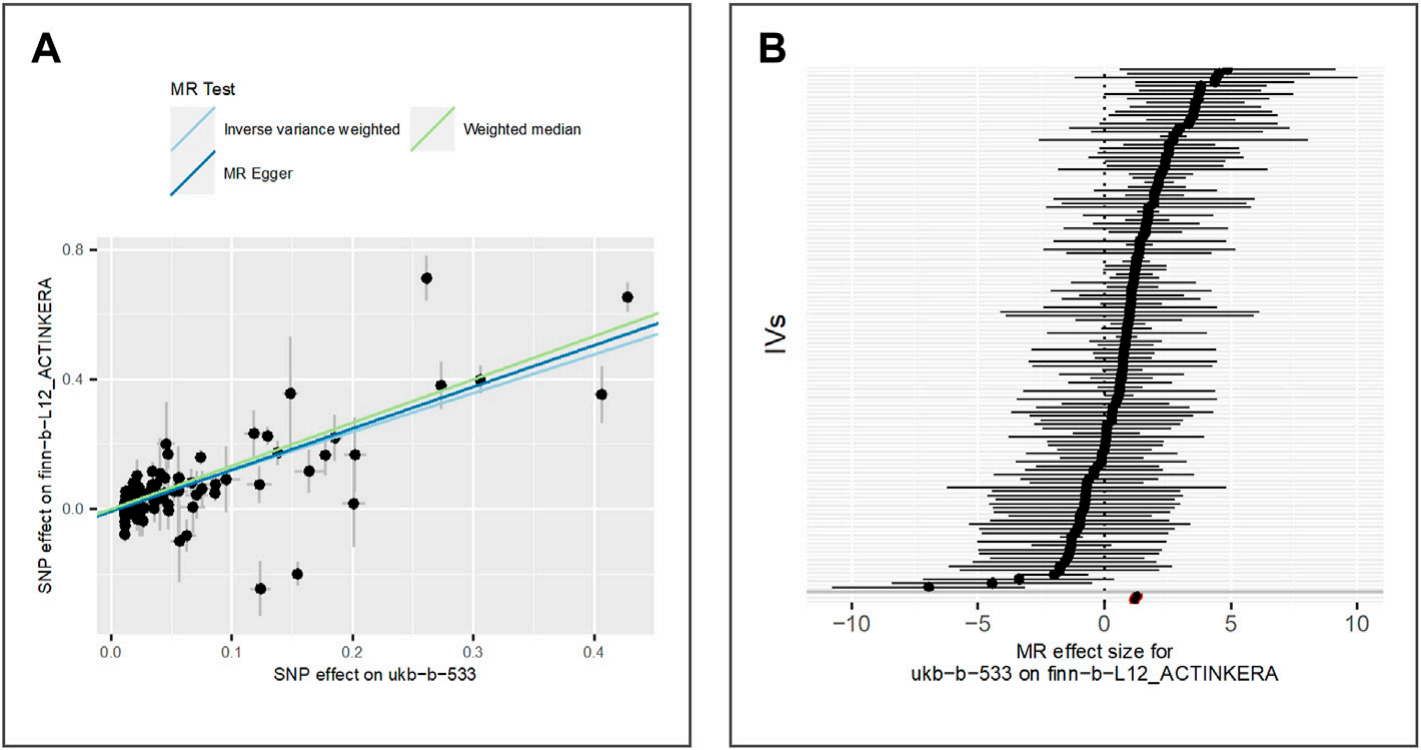
Comparison of MR methods and results associated with actinic keratosis. **(A)** Scatter plot reflects SNP effects on the actinic keratosis against SNP effects on the exposure; **(B)** forest plot represents the causal effect of exposure on the outcome using each SNP singly.

To analyze the effect of tanning response to sun exposure on actinic keratosis, we extract 125 IVs from IEU databased (ID: finn-b-L12_ACTINKERA). A total of 4,817 actinic keratosis cases and 213,273 controls of the European population were involved in the original GWAS. The tanning response to sun exposure is significantly associated with increased risk of actinic keratosis according to the results of IVW (beta = 1.19, *p*-value = 1.71E-41, adjusted *p*-value = 1.71E-40), MR Egger (beta = 1.28, *p*-value = 3.46E-22) and weighted median (beta = 1.33, *p*-value = 2.39E-45). The results are shown in Figure 2.

The Egger bias intercept is used to test the horizontal pleiotropy (intercept = -7.69E-03, *p*-value = 0.15) and the result indicates there is no horizontal pleiotropy. However, we find the presence of heterogeneity in the IVW analysis (*p*-value = 6.41E-26) and MR Egger analysis (*p*-value = 2.82E-25) using the Cochran Q test. Then we use the random effects model of IVW to estimate the effect of tanning response to sun exposure, and the results (beta = 1.19, *p*-value = 1.70558E-41) indicate there is still a positive correlation between exposure and actinic keratosis after considering the random noise. We performed a leave-one-out (LOO) sensitivity analysis to detect if the association was disproportionately influenced by a single SNP. We could see the LOO results in Supplementary Figure S1 and there was no bias caused by any single SNP.

### 3.2 Effect on seborrhoeic keratosis

**FIGURE 3:**
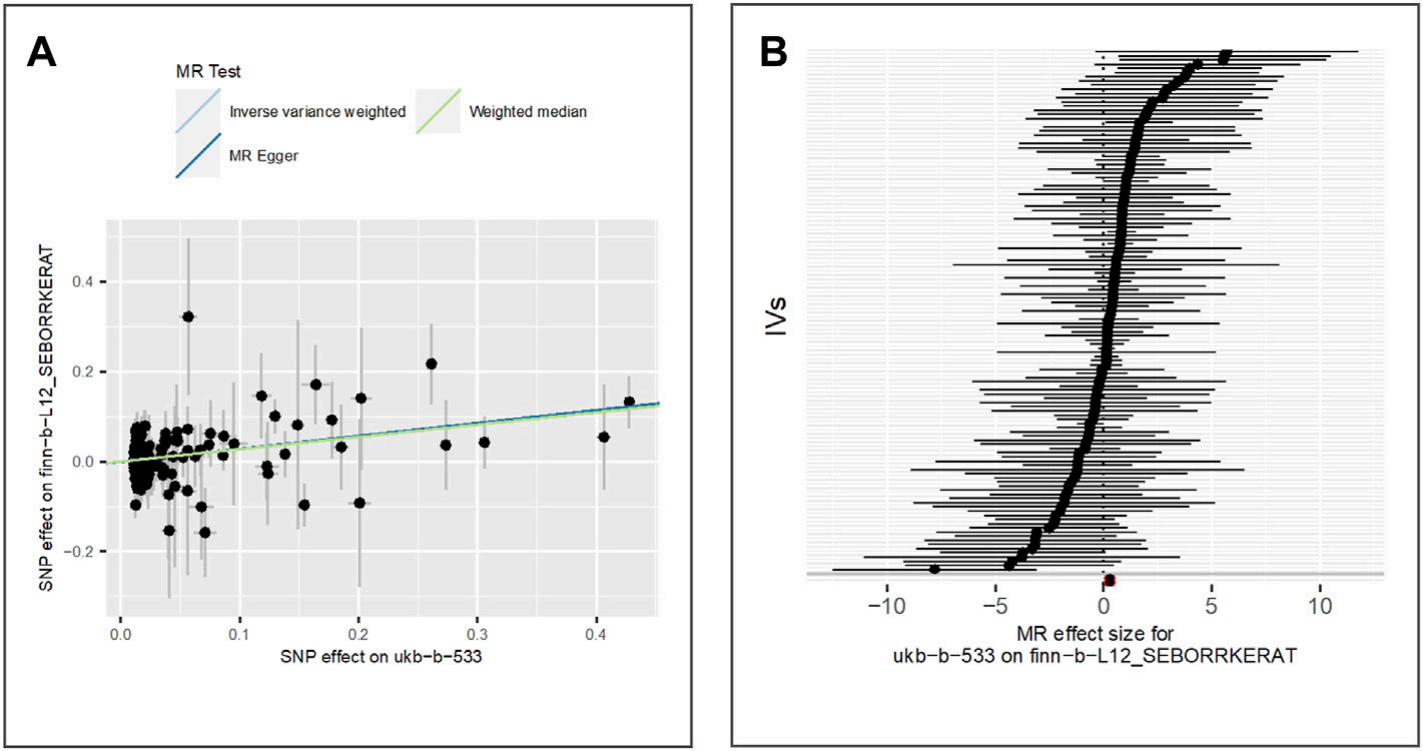
Comparison of MR methods and results associated with seborrhoeic keratosis: **(A)** scatter plot reflects SNP effects on the seborrhoeic keratosis against SNP effects on the exposure; **(B)** forest plot represents the causal effect of exposure on the outcome using each SNP singly.

We evaluate the effect of exposure on seborrhoeic keratosis using the GWAS dataset finn-b-L12_SEBORRKERAT. 2,434 cases and 207,482 controls of the European population were included in the GWAS. Also, we extract 125 valid IVs for downstream MR analysis. MR results of IVW (beta = 0.28, *p*-value = 5.94E-05, adjusted *p*-value = 2.97E-04), MR Egger (beta = 0.29, *p*-value = 1.06E-03) and weighted median (beta = 0.27, *p*-value = 1.30E-02) show evidence for the positive association between the exposure and seborrhoeic keratosis. Figure 3 shows the scatter plot for method comparison and forest plot indicating the MR effect of each IV.

The results of the Egger bias intercept (intercept: 5.50E-04, *p*-value = 0.90) show that there was no horizontal pleiotropy in the MR analysis. The results of IVW analysis (*p*-value = 0.30) and MR Egger analysis (*p*-value = 0.28) using the Cochran Q statistic show that there was no heterogeneity either. LOO analysis in Supplementary Figure S2 showed that results were not biased by a single SNP.

### 3.3 Effect on blepharochalasis

**FIGURE 4:**
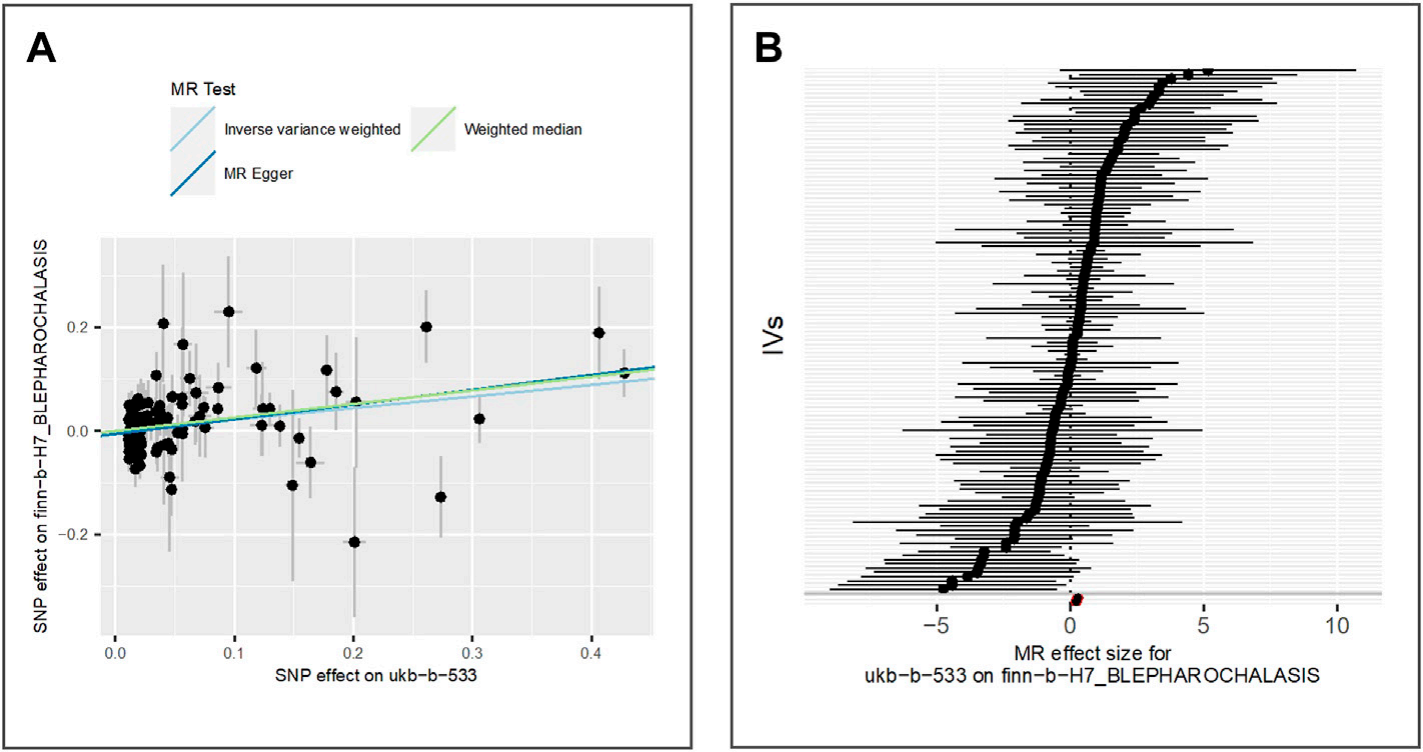
Comparison of MR methods and results associated with blepharochalasis: **(A)** scatter plot reflects SNP effects on the blepharochalasis against SNP effects on the exposure; **(B)** forest plot represents the causal effect of exposure on the outcome using each SNP singly.

To estimate the effect of tanning response to sun exposure on blepharochalasis, we extract 125 IVs from finn-b-H7_BLEPHAROCHALASIS GWAS dataset which is derived from a cohort of 4,135 cases and 203,231 controls of the European population. The results of IVW (beta = 0.22, *p*-value = 3.90E-04, adjusted *p*-value = 1.30E-03), MR Egger (beta = 0.28, *p*-value = 2.91E-04) and weighted median (beta = 0.26, *p*-value = 2.84E-03) (as shown in Figure 4) indicate that tanning response to sun exposure is positively associated with the risk of blepharochalasis.

The results of the Egger bias intercept (intercept: 5.45E-03, *p*-value = 0.16) show that there was no horizontal pleiotropy. The results of IVW analysis (*p*-value = 4.77E-03) and MR Egger analysis (*p*-value = 5.99E-03) using the Cochran Q statistic indicate the presence of significant heterogeneity. We applied the random effect model of IVW to re-estimate the MR effect, and the results under the IVW-random effect (beta = 0.22, *p*-value = 3.90E-04) indicate a statistically significant positive relationship between exposure and outcome considering the random noises. LOO analysis in Supplementary Figure S3 showed that results were not biased by a single SNP.

### 3.4 Effect on seborrhoeic dermatitis

**FIGURE 5:**
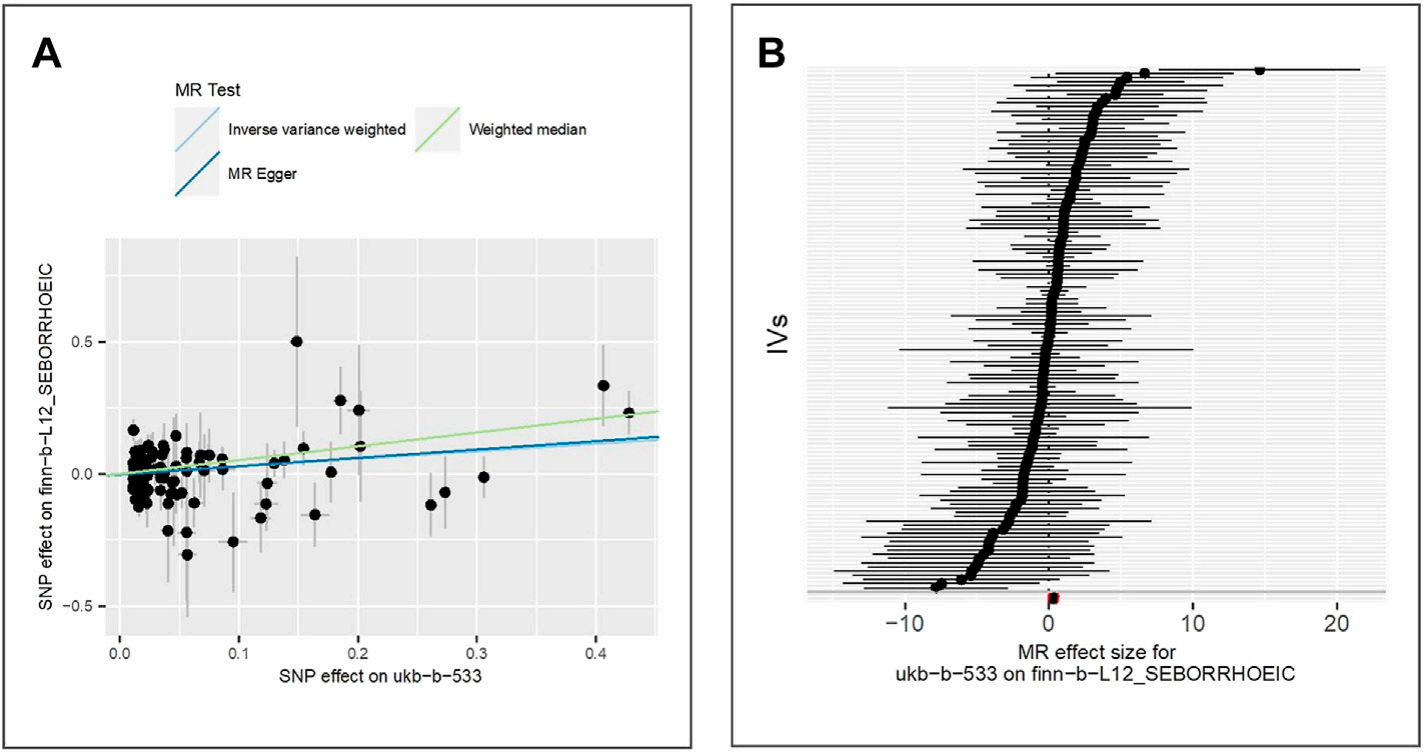
Comparison of MR methods and results associated with seborrhoeic dermatitis: **(A)** scatter plot reflects SNP effects on seborrhoeic dermatitis against SNP effects on the exposure; **(B)** forest plot represents the causal effect of exposure on the outcome using each SNP singly.

There are 1,253 cases and 198,740 controls of the European population in the GWAS of seborrhoeic dermatitis (IEU ID: finn-b-L12_SEBORRHOEIC). We extract 125 IVs to perform MR analyses. The results of IVW (beta = 0.29, *p*-value = 5.15E-03, adjusted *p*-value = 1.29E-02), MR Egger (beta = 0.32, *p*-value = 1.23E-02) and weighted median (beta = 0.52, *p*-value = 3.40E-04) (as shown in Figure 5), indicate positive significant association between tanning response to sun exposure and seborrhoeic dermatitis.

The results of the Egger bias intercept (intercept: 2.78E-03, *p*-value = 0.66) show that there is no horizontal pleiotropy. We perform heterogeneity analyses through IVW analysis (*p*-value = 5.44E-02) and MR Egger analysis (*p*-value = 4.93E-02) using the Cochran Q test. The IVW random effect results (beta = 0.29, *p*-value = 5.15E-03) show a significant positive relation between tanning response to sun exposure and seborrhoeic dermatitis. LOO analysis in Supplementary Figure S4 showed that results were not biased by a single SNP.

### 3.5 Effect on malignant melanoma of the skin

**FIGURE 6:**
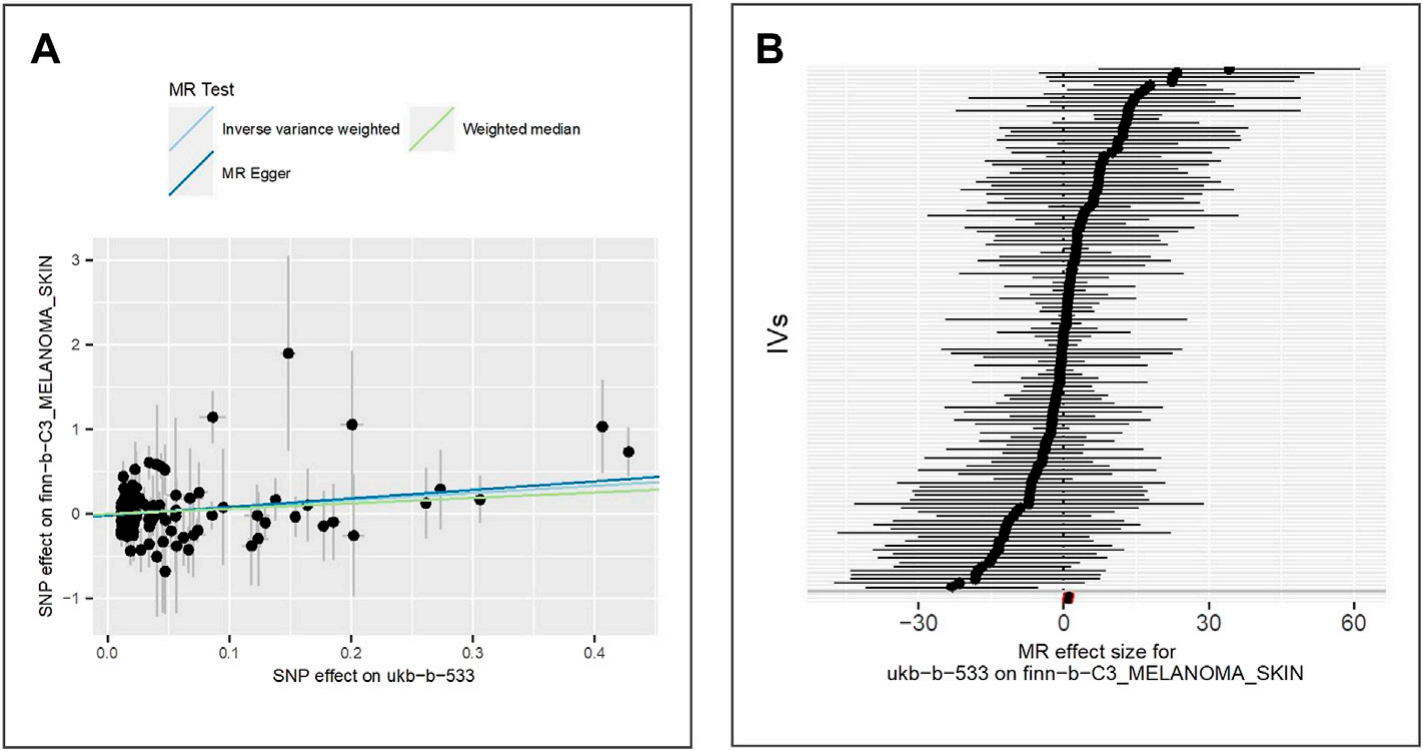
Comparison of MR methods and results associated with malignant melanoma of the skin: **(A)** scatter plot reflects SNP effects on the malignant melanoma of skin against SNP effects on the exposure; **(B)** forest plot represents the causal effect of exposure on the outcome using each SNP singly.

We extract the summary statistics from GWAS of malignant melanoma of skin (IEU ID: finn-b-C3_MELANOMA_SKIN) and find 125 valid IVs. We observe a positive association between exposure and the malignant melanoma of skin using all three methods, for example. IVW (beta = 0.83, *p*-value = 1.67E-02, adjusted *p*-value = 2.95E-02), MR Egger (beta = 1.01, *p*-value = 1.91E-02), and weighted median (beta = 0.63, *p*-value = 2.12E-01). The scatter plot for method comparison and forest plot indicating the MR effect of each IV is shown in Figure 6.

The results of the Egger bias intercept (intercept: 1.56E-02, *p*-value = 0.47) show that there was no horizontal pleiotropy. The results of IVW analysis (*p*-value = 0.177) and MR Egger analysis (*p*-value = 0.170) using the Cochran Q statistic neither indicate heterogeneity in the MR analysis. LOO analysis in Supplementary Figure S5 showed that results were not biased by a single SNP.

### 3.6 Effect on freckles

**FIGURE 7:**
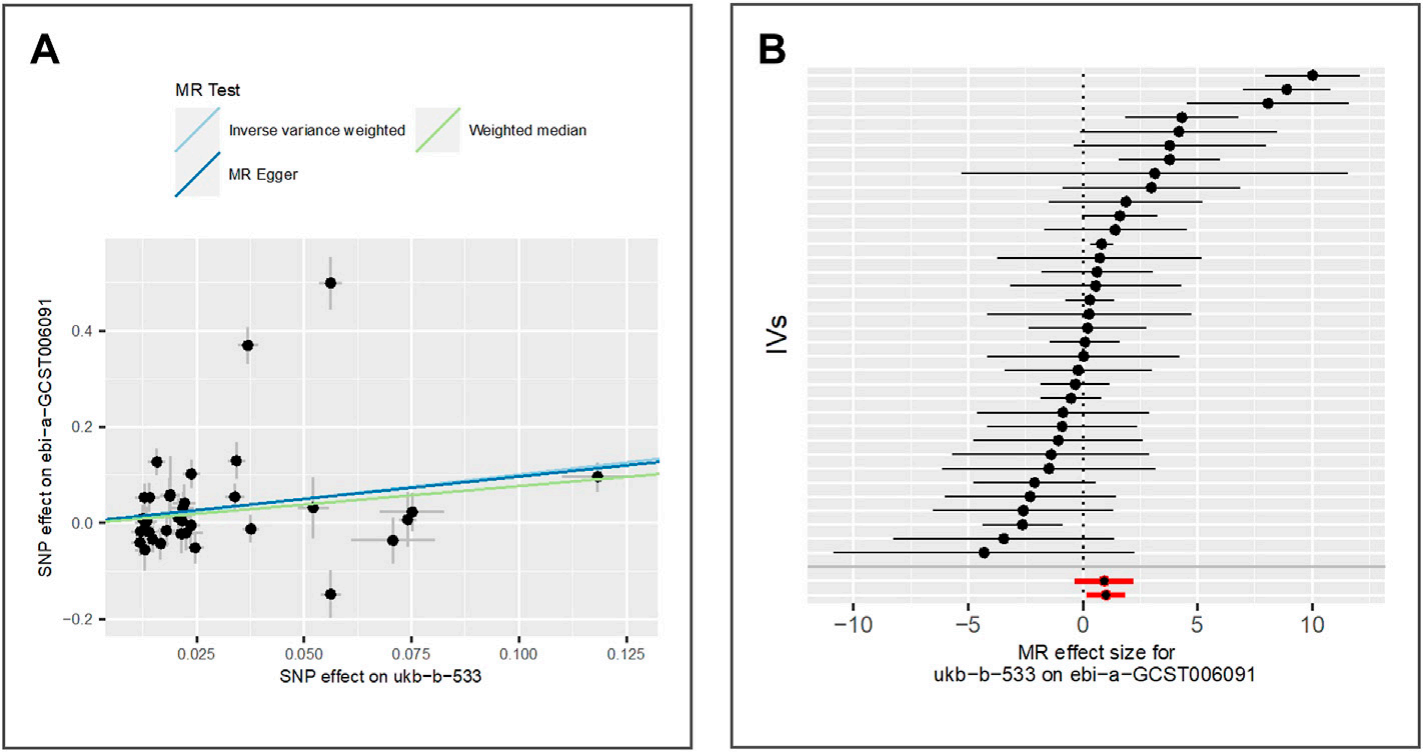
Comparison of MR methods and results associated with freckles: **(A)** scatter plot reflects SNP effects on the freckles against SNP effects on the exposure; **(B)** forest plot represents the causal effect of exposure on the outcome using each SNP singly.

We extract 35 IVs from GWAS of freckles (IEU ID: ebi-a-GCST006091). This GWAS included 7,148 freckles cases and 4,034 controls of the East Asian population. We find that tanning response to sun exposure is positively associated with elevated risk of freckles using the two measuring methods IVW (beta = 1.01, *p*-value = 1.77E-02, adjusted *p*-value = 2.95E-02) and weighted median (beta = 0.77, *p*-value = 1.02E-03), while the result of MR Egger is not significant (beta = 0.93, *p*-value = 1.69E-01). The scatter plot for method comparison and the forest plot indicating the MR effect of each IV is shown in Figure 7.

The results of the Egger bias intercept (intercept: 3.52E-03, *p*-value = 0.88) show that there is no horizontal pleiotropy in the MR study. The results of IVW analysis (*p*-value = 1.91E-30) and MR Egger analysis (*p*-value = 7.68E-31) using the Cochran Q statistic indicate that heterogeneity exists. We then re-analyze the association using the IVW random effect model, and the results (beta = 1.01, *p*-value = 1.77E-02) show a significant positive association between tanning response to sun exposure and freckles. LOO analysis in Supplementary Figure S6 shows that results were not biased by a single SNP.

### 3.7 The replication study

To replicate the associations found between the ease of skin tanning and skin diseases, we perform MR using independent outcome GWAS datasets derived from the UKBB. Five of ten skin diseases are available in the UKBB, including actinic keratosis, chalazion, malignant melanoma of the skin, melanocytic naevi, and seborrhoeic keratosisdata. The summary of relative GWASs derived from UKBB is detailed in Table 4. The MR results and sensitivity results are shown in Tables 5 and 6, respectively. We observe similar associations as in the discovery phase, indicating the robustness of our findings.

**TABLE 4:** Summary of the outcome GWAS in the replication study.

Outcome	ID in the IEU GWAS database	Population	Number of IVs	Number of	
				Cases	Controls
Actinic keratosis	ukb-d-L12_ACTINKERA	European	122	1,349	359,845
Chalazion	ukb-d-H7_CHALAZION	European	123	1,418	359,776
Malignant melanoma of skin	ukb-d-C3_MELANOMA_SKIN	European	127	2,534	358,660
Melanocytic naevi	ukb-d-D22	European	127	3,501	357,693
Seborrhoeic keratosis	ukb-b-501	European	78	1,957	461,053

**TABLE 5:** MR results between tanning response to sun exposure and five skin diseases in the replication study.

Outcome	IVW			MR Egger			Weighted median		
	Beta	se	*p*-value	Beta	se	*p*-value	Beta	se	*p*-value
Malignant melanoma of skin	8.11E-03	6.18E-04	2.13E-39	8.81E-03	7.27E-04	8.21E-23	8.81E-03	6.00E-04	8.78E-49
Actinic keratosis	3.46E-03	2.93E-04	4.64E-32	3.56E-03	3.48E-04	4.55E-18	3.52E-03	4.98E-04	1.39E-12
Melanocytic naevi	2.17E-03	6.87E-04	1.58E-03	1.88E-03	8.17E-04	2.28E-02	1.49E-03	8.11E-04	6.62E-02
Seborrhoeic keratosis	1.37E-03	4.38E-04	1.79E-03	1.45E-03	5.56E-04	1.09E-02	1.28E-03	6.01E-04	3.28E-02
Chalazion	2.01E-04	3.18E-04	5.27E-01	-2.00E-04	3.72E-04	5.92E-01	2.85E-05	4.35E-04	9.48E-01

Abbreviations: se, standard error; IVW, inverse variance weighted; MR, mendelian randomization; FDR, false discovery rate adjusted *p* value.

**TABLE 6:** The results of sensitivity analyses in the replication MR study.

Outcome	Horizontal pleiotropy			Heterogeneity					
	Egger intercept	se	*p*-value	IVWQ	IVWQ df	IVW *p*-value	MR Egger Q	MR Egger Q df	MR Egger *p*-value
Malignant melanoma of skin	−7.25E-05	4.09E-05	0.08	300.23	126	2.98E-16	292.88	125	1.67E-15
Actinic keratosis	−1.11E-05	1.95E-05	0.57	117.60	121	5.71E-01	117.28	120	5.53E-01
Melanocytic naevi	2.98E-05	4.60E-05	0.52	262.18	126	1.36E-11	261.30	125	1.17E-11
Seborrhoeic keratosis	−5.67E-06	2.29E-05	0.80	91.73	77	1.21E-01	91.66	76	1.07E-01
Chalazion	4.18E-05	2.09E-05	0.05	135.98	122	1.83E-01	131.63	121	2.40E-01

Abbreviations: se, standard error; IVW, inverse variance weighted; MR, mendelian randomization; Q, Cochran’s Q test estimate; df, Cochran’s Q test degrees of freedom.

## 4 Conclusion

Most skin changes are caused by sun exposure. To discover the causal relationship between sun exposure and tanning, as well as to develop effective therapies and preventative measures, we used a Mendelian randomization study to explore the causal association between tanning response to sun exposure and skin disease development based on multiple large-scale genome-wide association study datasets of the European population. Using Mendelian randomization studies, we found a positive correlation between tanning and skin diseases in the European population. There is a significant correlation between six skin conditions and tanning response to exposure to sunlight, including actinic keratosis, seborrhoeic keratosis, blepharochalasis, seborrhoeic dermatitis, malignant melanoma of skin, and freckles.

## Data Availability

The original contributions presented in the study are included in the article/Supplementary Material, and further inquiries can be directed to the corresponding author.

## Abbreviations

GWAS, Genome-wide association study; IVW, inverse-variance weighted; LSC, lichen simplex chronicus; MR, Mendelian randomization; MR-PRESSO, MR pleiotropy residual sum and outlier; SD, standard deviation; UKBB, United Kingdom Biobank.
