# Supplementary figures and images for: Evaluating the effect of tanning response to sun exposure on the risk of skin diseases through Mendelian randomization
Source: Front Genet. 2022 Sep 2;13:967696. doi: 10.3389/fgene.2022.967696 (PMC9478173; doi:10.3389/fgene.2022.967696)

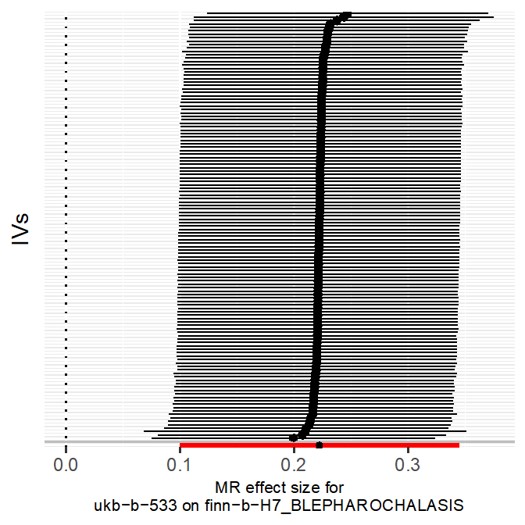

Supplement: Supplementary file 1 [file Image3.JPEG]

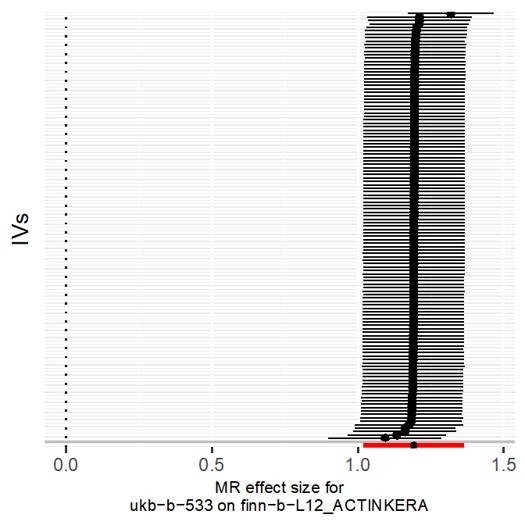

Supplement: Supplementary file 2 [file Image1.JPEG]

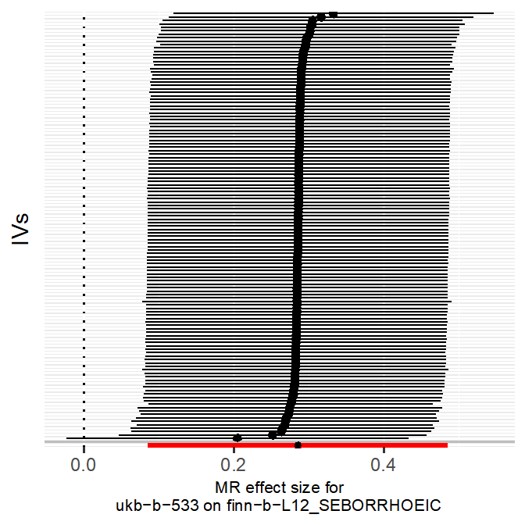

Supplement: Supplementary file 3 [file Image4.JPEG]

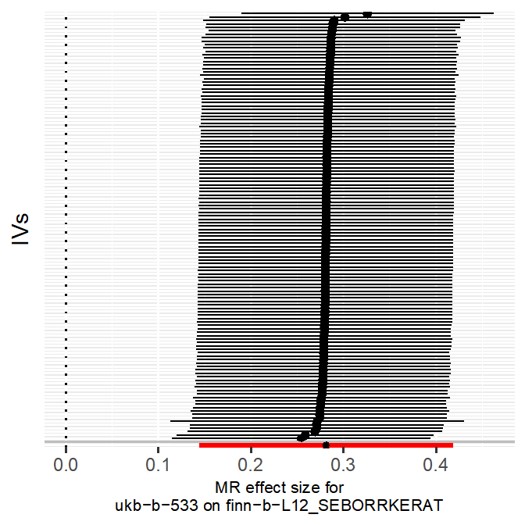

Supplement: Supplementary file 4 [file Image2.JPEG]

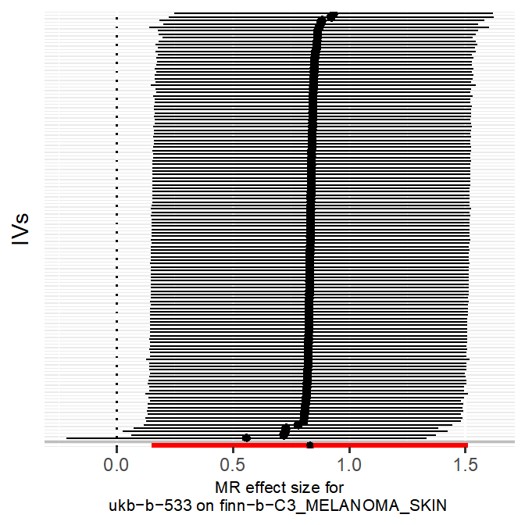

Supplement: Supplementary file 5 [file Image5.JPEG]

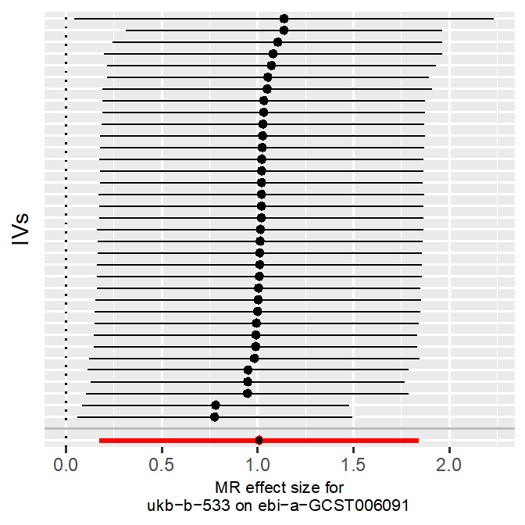

Supplement: Supplementary file 7 [file Image6.JPEG]
