# Supplementary material for: Evaluating the effect of tanning response to sun exposure on the risk of skin diseases through Mendelian randomization
Source: Front Genet. 2022 Sep 2;13:967696. doi: 10.3389/fgene.2022.967696 (PMC9478173; doi:10.3389/fgene.2022.967696)
Supplement: Supplementary file 6 [file DataSheet1.docx]

supplementary Figure S1.MR effect size for ukb-b-533 on finn-b-L12_ACTINKERA.

Supplementary FigureS2. MR effect size for ukb-b-533 on finn-b-L12_SEBORRKERAT.

Supplementary Figure S3. MR effect size for ukb-b-533 on finn-b-H7_BLEPHAROCHALASIS.

supplementary Figure S4. MR effect size for ukb-b-533 on finn-b-L12_SEBORRHOEIC.

supplementary Figure S5. MR effect size for ukb-b-533 on finn-b-C3_MELANOMA_SKIN.

supplementary Figure S6. MR effect size for ukb-b-533 on ebi-a-GCST006091.
